# Pregnant women's attitudes towards alcohol consumption

**DOI:** 10.1186/1471-2458-9-175

**Published:** 2009-06-05

**Authors:** Neil Raymond, Charlotte Beer, Cristine Glazebrook, Kapil Sayal

**Affiliations:** 1 Division of Psychiatry, School of Community Health Sciences, University of Nottingham, Nottingham, UK

## Abstract

**Background:**

There is uncertainty as to whether there is a safe threshold for drinking alcohol during pregnancy. We explored pregnant women's attitudes towards drinking alcohol in pregnancy and their attitudes towards sources of information about drinking in pregnancy following recent changes in UK government guidance.

**Methods:**

A qualitative study involving individual, semi-structured interviews with 20 pregnant women recruited from community organisations in the UK. Interview transcripts were analysed qualitatively using thematic analysis.

**Results:**

Most women found information and advice about safe levels of drinking in pregnancy confusing and lacking in evidence and detail. Although most women considered that there were risks involved with drinking in pregnancy and these perceptions influenced their behaviour, only six women reported abstinence. Women reported being influenced by advice from family and friends and their experiences of previous pregnancies. Many had received no individual advice from general practitioners or midwives relating to drinking during pregnancy.

**Conclusion:**

Pregnant women wished to take responsibility for their own health and make choices based on informed advice. In order to do so, they require clear and consistent advice about safe levels of drinking from policy makers and health professionals. This is an important issue as women might drink socially during their pregnancy.

## Background

Alcohol can readily cross the placenta and interfere with fetal development. High levels of alcohol consumption in pregnancy are associated with harmful effects such as fetal alcohol syndrome [1]. In recent years, the debate has focused on the safety of light drinking during pregnancy although the research findings are inconclusive [2-8]. Some studies [6,7] suggest an association between light drinking and childhood behavioural problems but a systematic review found no consistent evidence of adverse effects across a range of outcomes [5]. More recently, a large epidemiological study also found no evidence of harmful effects on child behaviour or learning [8]. Hence, a safe threshold for drinking in pregnancy has not been conclusively established [9-11]. The proportion of women of child-bearing age in the UK who drink over 14 units of alcohol per week (one unit is equivalent to one glass and contains 8 grams of alcohol) has increased in recent years [12]. Consequently, the fetus is most likely to be exposed to alcohol in the first trimester, before pregnancy recognition [13]. These findings suggest that alcohol consumption during pregnancy is an important public health issue.

Government advice regarding alcohol consumption in pregnancy varies internationally. In many countries, including the US, Canada and France, complete abstinence is recommended. Until recently, the UK Department of Health guidance [14] was that pregnant women should limit themselves to 1–2 'units' of alcohol once or twice a week and avoid getting drunk. In May 2007, the guidance was adjusted to recommend abstinence [15]. This primarily reflected the greater clarity of a 'no drinking' message rather than the research evidence [10]. However, the guidance also stated that, if pregnant women choose to drink, they could drink up to the previously recommended limits. The implications that this guidance will have on pregnant women's drinking behaviour are unclear.

Further uncertainty is created by the differing guidance provided by other UK health organisations. The Royal College of Obstetricians and Gynaecologists [16] have recommended a limit identical to the previous Department of Health guidance. In contrast, the British Medical Association [3] expressed concern that government guidance and drinking limits could be misinterpreted and advised pregnant women to avoid alcohol. This uncertainty was highlighted when National Institute of Clinical Excellence (NICE) draft clinical guidelines for antenatal care initially suggested that pregnant women should limit their alcohol intake to less than 1.5 units per day [17]. However, following consultation, the recommendation was revised to maintain greater consistency with the Department of Health guidance [18]. These discrepancies in advice as well as research findings have generated vast media coverage, often involving conflicting opinions. Pregnant women are likely to receive information from sources such as news reports, magazine articles, online information and advice from health professionals, family, and friends. Inconsistent reporting of information could lead to considerable uncertainty and anxiety [19] for pregnant women about whether it is safe to drink and which guidelines to follow.

Most research exploring pregnant women's attitudes towards drinking in pregnancy has been quantitative [20,21]. These studies have found that pregnant women are aware that alcohol can harm their unborn babies but most believed that some alcohol intake during pregnancy was acceptable [20,21]. Such research suggests women may be reluctant to follow guidance recommending complete abstinence.

A qualitative approach could provide a richer understanding of women's attitudes towards alcohol consumption during pregnancy and can give insight into previously unexplored areas [22]. A better understanding of women's attitudes towards sources of information and government guidance is essential if health information is to be made clinically relevant and credible for them. In this exploratory study, we aimed to explore pregnant women's attitudes towards alcohol consumption during pregnancy and their attitudes towards sources of information and advice about drinking in pregnancy.

## Methods

### Sample and Procedure

In late 2007, a few months after the Department of Health guidance had changed, we purposively sampled a group of women who engaged in antenatal care in order to explore their range of knowledge and attitudes. We anticipated that some of these women might drink socially during their pregnancy [6,8]. Pregnant women in Nottingham and London were recruited from a range of community organisations including Sure Start Children Centres (antenatal input is also provided here), National Childbirth Trust antenatal groups and mother and toddler groups. Details about the study were sent to these community organisations. Co-ordinators of the organisations that agreed to take part distributed information packs about the study to pregnant women. These packs included the study Information Sheet, Consent Form and contact details form. All women were given the option of individual telephone or face-to-face interviews. We used individual interviews as this allowed participants anonymity, confidentiality, and the opportunity to express themselves freely in a non-threatening forum. Ethics approval was received from the University of Nottingham Medical School Research Ethics Committee.

Twenty women agreed to participate within the study period and all requested telephone interviews. These semi-structured interviews were carried out by the same interviewer and lasted between 20–40 minutes. Informed consent was obtained prior to the interview and participants returned their signed consent forms by post. The interviews were audio recorded and transcribed verbatim. Women were asked open questions using a topic guide covering the following areas: information received about drinking during pregnancy; the influence of health professionals, friends, family, and the media on their drinking; their attitudes towards government and other advice; and their views about drinking during pregnancy and available health information. This provided standardisation between the individual interviews. During the interview, women were also asked some questions about their demographic status to establish sample characteristics and some closed questions about their current and pre-pregnancy levels of alcohol consumption and their knowledge of Government advice. Responses to the closed questions are reported descriptively in the Participant Characteristics.

### Data Analysis

The interview transcriptions were analysed using thematic analysis [23]. Theme analysis was chosen for its flexibility which allows a full exploration of the data without the constraints of methods such as interpretative phenomenological analysis or grounded theory analysis which are more theoretically bounded [24]. With the collected data, theme analysis enabled the identification and description of barriers and facilitators to drinking in pregnancy. Theme analysis allows themes to be produced both deductively where they are generated by the researcher based on previous theory and research literature before analysis and inductively, from the raw data itself [25]. Initially, the transcripts were thoroughly read by N.R. to become familiarised with the ideas and attitudes expressed. The transcripts were then systematically searched using NVivo7 (QSR International) to identify all codeable moments. These codeable moments were grouped together to create themes which were reviewed, debated and refined methodically by three researchers (N.R., C.S., K.S.) resulting in eight final themes. Four themes were identified inductively and four were generated deductively based on past theory and research.

Once the themes were identified, a thematic code for each was developed. Each thematic code satisfied Boyatzis' five elements including: a conceptually meaningful label, a definition, a description of how to know when the theme occurs, a description of any qualifications or exclusions to the application of the theme, and examples of positively and negatively coded extracts from the data [23]. These thematic codes were put together to form a code book. In order to establish the reliability of the themes, an experienced independent researcher used the code book to code a sample of 21 extracts selected to represent all eight themes. Inter-rater agreement was high with concordance for 18/21 (86%) extracts.

## Results

### Participant Characteristics

The women's ages ranged between 23 and 40 years (median 33 years). They were between 12 and 40 weeks pregnant (median 26 weeks). Four of the women had two children and seven women had one child. All participants were either married or co-habiting with a partner, educated to 'A-level' or beyond and had drunk alcohol before the pregnancy (the majority were married, had a university education and usually consumed up to 1–2 drinks per week before the current pregnancy). All but one reported a reduction in their alcohol consumption after pregnancy recognition, six abstaining completely. In terms of patterns, three women described consuming up to 1–2 drinks in total, seven up to 1–2 drinks per month, three up to 1–2 drinks per week, and one more than 1–2 drinks per week. Participants described safe levels of alcohol consumption during pregnancy as ranging from no alcohol at all, through one glass a day, to four glasses a day.

Half the women did not recall receiving any specific advice from health professionals about drinking during their current pregnancy. However, some mentioned being given written information by the general practitioner (GP) or midwife. Reported advice received on safe drinking levels ranged from abstinence to one to four units a week. Some women also mentioned that friends had been recommended different limits. Knowledge of the government advice varied. Thirteen women were aware that the advice had changed. Although eight of these women correctly recalled this, none were confident that they were correct.

### Themes: barriers and facilitators to drinking in pregnancy

Eight themes were identified which related to the study aims (see Table 1). These all reflected barriers and facilitators to drinking in pregnancy.

**Table 1:** Themes

**Themes**	**Method of generation**
Evaluation of risks	Deductive
Unborn child has precedence	Inductive
Influence of previous and other pregnancies	Deductive
Individual differences	Inductive
Facilitators to drinking in pregnancy	Inductive
Influence of confusing or unclear advice	Deductive
Attitudes towards available advice: Advice lacks reasons, evidence or sufficient detail	Deductive
Taking responsibility for own health	Inductive

### The influence of evaluation of risks on drinking in pregnancy

Prompted by previous research findings [20,26] that women were aware of the potential harm to their unborn baby of drinking during pregnancy, we explored how women evaluated the risks of drinking. Participants had assessed or considered the risk to themselves or to their unborn child. Perceived risk often influenced their drinking behaviour. If they thought that there was a high risk involved, they tended to completely abstain from alcohol or limit themselves to a very small amount. Women who were uncertain about possible risks erred on the side of caution.

*"I go for the safety aspect, so because I'm not 100% sure, I just completely abstain to be on the safe side." *(Interview 10, no children)

Similarly women who found the given information inconsistent tended to be more cautious.

*"Because there is so much conflicting information, I do think that the way to go is if you don't drink any then there's no risk." *(Interview 8, no children)

In contrast, women who thought that there was a low risk described more relaxed attitudes.

*"If you drink in moderation and you're sensible, then I don't think it affects the fetus and if it has a relaxing effect on you, then I don't see there's any harm." *(Interview 14, two children)

### Unborn child has precedence over drinking in pregnancy

This theme emerged inductively from the data and was related to the above theme. Participants described how their unborn child's interests were first and foremost in their minds. These women revealed feeling an obligation to protect their child's health and safety.

*"I've got to think of my child, I've got to put them first." *(Interview 7, no children)

This view often took priority over any wish to drink alcohol while pregnant. Women therefore limited their drinking or completely abstained from alcohol during their pregnancy. In addition, a common view was that the period of time involved was manageable if the child's best interest was at heart.

*"It is only 9 months which isn't very long... So it's not that long really when you've got to think about somebody else's life." *(Interview 7, no children)

### Influence of previous and other women's pregnancies on drinking in pregnancy

Other researchers [27] have found an association between previous pregnancy experiences and drinking behaviour and we explored this theme. Women recalled their own previous experiences of drinking in pregnancy or the pregnancies of friends and family members and the lack of any harmful effects on the outcome.

*"I drank a little bit with my first child and I carried on doing that with my second and third pregnancies. My first child is absolutely fine." *(Interview 2, two children)

*"I'm listening to the older generation as well and they used to listen to those old guidelines and most children are OK and weren't affected by one or two units a week if they chose to have it." *(Interview 7, no children)

These experiences were mostly reassuring and these women did not attribute drinking in pregnancy to any harm to the child. Often, they were used as an example to justify the safety of drinking in pregnancy. However, family experiences were also described that negatively associated drinking in pregnancy with the child's health.

*"She was drinking because they never realised she was pregnant. And her son does have learning disabilities. Whether that's connected or not, I don't know." *(Interview 1, two children)

### Need to respect individual differences

In relation to the previous theme, the theme of individual differences emerged inductively from the data. Women acknowledged how alcohol can vary in its effects on different people and were aware of individual differences between women.

*"And its one of those things where everybody's different so you don't know whether a little bit will affect you or have no effect whatsoever." *(Interview 9, no children)

The recognition of individual differences was often associated with advocating drinking behaviour that felt comfortable for each individual.

*"I think you have to do what you feel is right for you." *(Interview 13, two children)

### Facilitators to drinking in pregnancy

An unexpected theme which emerged inductively from the data was the benefits of drinking. Women described drinking as being beneficial with regard to stress relief and relaxation and that the positive effects of alcohol outweighed the possible risks.

*"I just know that it gives me just that total relaxation feeling... which I guess could outweigh the fact that you're having alcohol." *(Interview 6, one child)

*"I also think it helps when you're really stressed out, emotionally up and down... as you are during pregnancy, occasionally have a glass of wine." *(Interview 2, two children)

### Influence of confusing or unclear advice on drinking in pregnancy

In anticipation that women would find current information unclear, we looked for this theme. The advice on drinking during pregnancy was described as being confusing, unclear or lacking sufficient detail.

*"Some books say you should avoid it all together and others say it's OK to perhaps have one glass. So even in the pregnancy books you get a confusing message." *(Interview 1, two children)

Concern was expressed about the conflicting advice.

*"It's very difficult to feel very reassured with any of the advice because everything conflicts so much. So... it has been very difficult." *(Interview 13, two children)

There was a call for the advice given from health professionals, government and other sources to become clearer and more consistent, in line with advice and information regarding smoking.

*"I think midwives should be a lot more clear about it. The info given to women in the big pack of info, when you go to your first visit, maybe that should be a little more clear." *(Interview 20, no children)

*"I mean they are very clear on smoking but not drinking." *(Interview 10, no children)

More specifically, the term 'units' used to describe measures of alcohol in government guidelines and by health professionals was regarded as being misunderstood or confusing.

*"Especially if you're drinking at home, it can be difficult to estimate how much a unit actually is." *(Interview 9, no children)

Clear advice was deemed to be particularly important for pregnant women who were less knowledgeable.

*"They need to sort it out really, I mean I know what's right and wrong, but if somebody else isn't that well educated and things like that, then the media is what they listen to, then they're going to listen to that kind of thing." *(Interview 7, no children)

### Attitudes towards available advice: Advice lacks reasons, evidence or sufficient detail

Given that a recent systematic review [5] had found no consistent evidence for adverse outcomes from light drinking during pregnancy, this theme was explored deductively. Existing advice and information was felt to lack legitimate justification, supporting research evidence or adequate detail. They suggested a need to deal with these shortcomings and provide more detailed information for pregnant women.

*"And the first time you are pregnant you do get a pregnancy book which goes into diet and I think it mentions it in there but I don't think there is enough said about why you shouldn't drink." *(Interview 11, one child)

Often pregnant women were asked about their alcohol consumption in consultations with health professionals but not given advice. In addition, where advice about alcohol was given by midwives and GPs, this was not considered to be sufficiently informative.

*"So there's not really much in the literature that you get from the midwife. They just tell you not to drink and they don't tell you why." *(Interview 11, one child)

### Taking responsibility for own health

An interesting theme to emerge inductively from the data was women's desire to take responsibility for their own health. Women wanted to make individual choices and take control over decisions about their own health.

*"I think it has to be everyone's individual decision certainly." *(Interview 5, one child)

Participants felt that women who wished to drink more than advised could make that decision and should take responsibility for their actions. In addition, women demonstrated concern that the government and public services were attempting to control how pregnant women acted and were making choices on their behalf.

*"I think we're all responsible enough to... I think there is too much bureaucracy and too much red tape around things." *(Interview 14, two children)

Women felt they were not given the opportunity to make an informed decision about their own health and the health of their unborn child. They recognised the need for more informative advice in order for women to take responsibility for themselves.

*"I generally feel that women should be given the information about... what is known, and the risks etc. and then left for them to make up their own minds." *(Interview 9, no children)

## Discussion

This qualitative study revealed diverse attitudes towards alcohol consumption during pregnancy among well-educated women. Women were found to have differing views on the risk associated with drinking alcohol and this influenced their consumption of alcohol. Their evaluation of risk was hindered by the availability of often conflicting advice from government guidelines, health organisations and the media. In addition, women often reported that they had not received individual advice from health professionals. This may have encouraged women to rely on anecdotal advice from family and friends, and experiences of past pregnancies. However, many women reported that they wanted official advice and guidelines.

In keeping with previous research, women had considered the potential risks to their unborn child from drinking alcohol during pregnancy and this often affected their attitudes towards drinking [26]. Higher perceived risk is associated with lower alcohol consumption in pregnancy [27]. Moreover, in keeping with surveys carried out in other countries, most women in our sample reported they had reduced their alcohol consumption following pregnancy recognition [26,28]. However, almost three quarters had consumed alcohol while pregnant which is in keeping with UK national statistics [29]. Their attitudes toward drinking alcohol during their current pregnancy were often influenced by experiences of other pregnancies, perhaps through evaluation of risk [27]. Many women recalled their levels of drinking in their previous pregnancies or those of family and friends and felt there had been no adverse influence on the child's health. Interestingly, some women described positive effects of drinking in pregnancy such as stress relief and often mentioned missing alcohol if they had reduced their consumption. As antenatal anxiety is a risk factor for child mental health problems [30], some women might perceive that the possible benefits of drinking during pregnancy might outweigh the potential risks. Furthermore, since our data were collected, there has been recent media interpretation and reporting of findings from a large study that drinking 1–2 glasses of alcohol a week during pregnancy could boost a child's intelligence and behaviour by the age of three years [8]. This is likely to have added to the uncertainty and confusion for pregnant women in the UK.

We also explored women's attitudes towards sources of information relating to alcohol consumption in pregnancy. Although most women were aware of some form of advice or information about this, their knowledge of the source and content of the information and their attitudes toward the advice differed considerably. Many women reported conflicting information from the media. Unsurprisingly, common views were that the information was confusing, unclear, or lacking in sufficient detail. In keeping with findings from other countries, health professionals reportedly offered limited or inconsistent advice to pregnant women, often not raising this issue at all [20,21,31]. A possible explanation is that health professionals are not certain whether abstinence from alcohol in pregnancy is necessary and that they are also similarly confused [32]. However, information and advice from health professionals are regarded as influential and persuasive and participants reported that more would be welcomed [21,33]. Furthermore, since there is clear evidence that alcohol has negative effects on infant development if consumed in sufficient quantity [1], it is particularly concerning that ambiguity in terms of safe limits may have deterred health professionals from offering any advice about limiting alcohol intake. This is an area that warrants further research.

### Methodological Issues

The purpose of this qualitative study is to provide information on women's attitudes towards alcohol consumption in pregnancy. As with other qualitative research, there are questions of information bias, reliability of results and validity of explanations. Different approaches to analysis may have yielded some differences in the findings. Attempts to reduce these effects included using a consensus approach to identifying themes with multiple researchers and checking inter-rater reliability for these themes. Although face-to-face interviews have the advantage of eliciting non-verbal information, telephone interviews were practical and conveniently fitted around the participants' lives. In addition, although women may have been more open in a relatively anonymous telephone interview compared to a face-to-face interview at an unfamiliar location, the direction of effect in terms of the confiding of sensitive information remains unclear [34]. For example, worry or guilt did not emerge as a theme in the study. Although the information given may have been influenced by the interviewer being male, the direction of influence is unclear. A range of diverse views were elicited, suggesting that it is unlikely that researcher bias had a major effect.

The findings from this exploratory study should be regarded as preliminary. Information about advice received about drinking during pregnancy was ascertained using closed questions and hence was not part of the thematic analysis. In terms of the sample, we were not able to collect information about why women chose not to participate in the study. We recruited from a range of groups in order to increase variation within the sample. Although we did not obtain information on their ethnicity or employment status, it is likely that sampling through groups may bias the sample towards more socially integrated women. Indeed, the majority of participants had a university education and were married and so their attitudes and drinking levels may differ from other women. For example, older, more highly educated mothers with children are more likely than other women to drink lightly during pregnancy [6,8,26]. Hence the issue of light drinking is salient. Despite our relatively homogeneous sample, women represented in this study reflect an important group. Exploring the views of these women provided important insights into their attitudes towards drinking during pregnancy and current advice and their desire for further information.

### Future research

In order to explore a broader range of knowledge and attitudes towards alcohol and influential sources of information, future research should incorporate more diverse samples. Our findings can provide the basis for a larger quantitative survey in the UK to assess pregnant women's attitudes towards alcohol and their drinking behaviour. It would also be useful to formally investigate how information is presented in the media and is provided to pregnant women by health professionals. Our study highlights the need for a similar study with GPs and midwives in order to establish their views about alcohol use in pregnancy and the provision of information. This may provide an explanation for women's perceptions of only limited advice being provided by health professionals. Comparative studies from other countries would enable cross-national comparisons of attitudes and perceptions of advice and drinking during pregnancy.

### Clinical and Policy Implications

Many women described the importance of personal choice in relation to health decisions in pregnancy. They also called for individual advice from health professionals and felt they would have found this useful. As alcohol consumption prior to pregnancy recognition is a common occurrence [13], health professionals should be aware that many women may seek reassurance.

Government policy makers and health professionals should recognise the importance of these issues to women who might drink socially during their pregnancy, especially as research on safe levels of drinking in pregnancy remains inconclusive. Our findings suggest that they need to ensure that pregnant women receive accurate and consistent information on which they can base decisions and take responsibility about their healthcare. They should be aware that women consume alcohol for a range of reasons, including relaxation, and advice needs to account for women who plan to continue to drink during pregnancy. The challenge for policy makers is to provide clear, consistent and credible information for both pregnant women and health professionals in contact with them.

## Conclusion

Pregnant women wished to be able to make fully informed decisions and to take responsibility for their own health. They felt that, at present, the available advice and guidelines lacked explanations and supporting evidence. Such findings are important, given that women reported being influenced by perceptions of risk and that many put their unborn child's needs first. Pregnant women require clear and consistent advice about safe levels of drinking from policy makers and health professionals.

## Declaration of Competing interests

The authors declare that they have no competing interests.

## Authors' contributions

We confirm that all authors fulfil the criteria for authorship. All authors contributed core ideas and to writing the paper. NR recruited the sample, carried out the interviews, and helped to draft the manuscript. CB participated in the design and co-ordination of the study, data analysis, interpretation of the findings, and writing the paper. CG participated in the conception of the study, interpretation of the findings, and writing the paper. KS had the original concept, supervised data collection and the co-ordination of the study, participated in the data analysis and interpretation of the findings, and drafted and finalised the paper. All authors read and approved the final manuscript.

## Pre-publication history

The pre-publication history for this paper can be accessed here:


